# Eyewitness testimony by individuals who stammer: Evidence, experience and perceived credibility

**DOI:** 10.1111/bjop.70014

**Published:** 2025-07-29

**Authors:** Katie Maras, Sohee Park, Patrick Grafton, Jasmin Peat, Navyaa Toshniwal, Alice Haigherty, Kevin Guo, Monty Franks, Hannah Goodwin, Victoria Grau Sainz, Amaira Sharma, Alisa Fridman, Luke Gordon‐Ellis, Kirsten Howells

**Affiliations:** ^1^ Department of Psychology University of Bath Bath UK; ^2^ STAMMA London UK

**Keywords:** credibility, jurors, language disorder, perceptions, recall, stammering, testimony, witness

## Abstract

Stammering may impede an individual's eyewitness testimony and reduce jurors' perceptions of their credibility through a complex interplay of bio‐psycho‐social factors. However, no research to date has explored this. Three co‐produced, mixed‐methods studies are reported, investigating the evidential quality, lived experiences and perceived credibility of people who stammer (PWS) as witnesses. In pre‐registered Study 1, PWS recalled as much correct information as non‐stammering witnesses overall. However, during the free – but not cued – recall interview phase, PWS provided fewer correct details. A reflexive thematic analysis of participants' post‐testimony reflections captured how PWS experienced a cyclical relationship between communicative pressure, anxiety over listener misperceptions and stammer severity, which they navigated either by employing avoidance strategies at the expense of testimony or by speaking through their stammer. In pre‐registered Study 2, mock jurors rated PWS as less confident yet more likeable and trustworthy than non‐stammering witnesses. In Study 3, providing jurors with information about stammering further improved their likeability and trustworthiness but had no impact on perceived confidence. Findings provide new insight into communication disorders in legal contexts – and the unique challenges faced by PWS in particular – demonstrating the need for systemic accommodations and targeted training for legal professionals.

## BACKGROUND

Navigating the criminal justice system (CJS) is challenging for any witness, but it can be especially difficult for those with speech, language and communication difficulties (Learoyd & Bryan, 2023). These challenges arise, in part, from the significant communication demands imposed during police interviews and court proceedings (Bagnall & Maras, 2025). Furthermore, case outcomes rely heavily on juror decisions that are significantly influenced by the presentation of individuals who testify (Bornstein & Greene, 2011). Juror assessments of credibility are often (erroneously) influenced by factors such as speech disturbances (e.g. hesitations, pauses and slower speech rate), emotional expression, eye contact, behavioural quirks, perceived confidence, likeability and trustworthiness, all of which are linked to an individual's communication and expression (e.g. Chalmers et al., 2022; Cooper et al., 2014; Jules & McQuiston, 2013; Neal & Brodsky, 2008; Vrij, 1998; Vrij & Fisher, 2020).

One group who, anecdotally, are suggested to face disproportionate challenges are people who stammer (PWS; Pritchard & Short, 2022). Stammering (also sometimes referred to as stuttering) is a speech fluency disorder that affects approximately 5% of the population at some point across their lifespan (Yairi & Ambrose, 2013). Stammering is characterized by difficulty in producing a smooth flow of speech, which typically manifests in core speech behaviours (e.g. elongations of syllables, hesitations and tense blocks), secondary behaviours (e.g. facial tension and excessive blinking), and cognitive and emotional features (e.g. speaking anxiety and stammering anticipation) (American Psychiatric Association, 2013; Jackson et al., 2015; Maguire et al., 2012). These features can significantly impact communication, with individuals often limiting their speaking or changing their syntax and engaging in behaviours such as foot tapping and eye closing to work through moments of stammering (Bricker‐Katz et al., 2013; Ward, 2008).

Stammering may impede effective communication in legal settings through a complex interplay of bio‐psycho‐social factors (Constantino et al., 2022). Providing testimony is stressful and anxiety‐provoking for most witnesses (Fairclough, 2024), which may in turn impair speech in an otherwise fluent speaker (Buchanan et al., 2014). However, giving testimony is likely to be disproportionately stressful and detrimental to the communication abilities of PWS (Parsons et al., 2022). Although stammering is not caused by anxiety or stress, such emotions often increase the frequency or intensity of stammering, particularly in high‐stakes interpersonal situations where communicative pressure is higher (Guitar, 2013; Parsons et al., 2022). This exacerbation may be partly due to the disruption these emotions cause to the motor control pathways responsible for speech fluency (Alm, 2004; Sander & Osborne, 2019).

Anticipating negative reactions from listeners contributes to the anxiety and speech disfluency PWS experience during communication exchanges (Boyle, 2018; Messenger et al., 2004; Tichenor & Yaruss, 2018). PWS are often perceived to be less competent and confident than those who do not stammer (Connery et al., 2020) and they encounter (and expect to encounter) negative responses to their speech (Connery et al., 2020; Guntupalli et al., 2007). Acute awareness of these assumptions leads many PWS to remain constantly vigilant to how they are being perceived when communicating (Bricker‐Katz et al., 2013), which can lead to heightened anxiety surrounding speaking and avoidance of certain situations and trigger words (Iverach et al., 2011). This hypervigilance is especially pronounced in interactions with public authorities, where some PWS fear being perceived as suspicious or dishonest (Wunderlich et al., 2023).

Resultantly, many PWS report prioritizing fluency at the expense of accurately articulating their intended message (Constantino et al., 2017; Parsons et al., 2022; Yarzebinski, 2022); for example, by using avoidance or coping strategies (e.g. swapping words or finding a different way to say something, saying less, or choosing not to participate in certain situations at all) to minimize their risk of stammering (Constantino et al., 2022; Grisham et al., 2015; Jackson et al., 2015, 2021). While these strategies may conceal overt stammering, they can undermine communication, leading to less precise word choices, abandoned speech and convoluted phrasing (Bricker‐Katz et al., 2013; Connery et al., 2020; Constantino et al., 2022). The cognitive burden of employing these strategies may further impact upon the content of communication (Iimura et al., 2016; Sander & Osborne, 2019); in bilingual speakers, for example, increased cognitive load from language‐switching reduces recall accuracy and self‐perceived credibility (Akehurst et al., 2018; Alm et al., 2019; Hu & Naka, 2023). It is possible, therefore, that the completeness and/or accuracy of the testimonies of PWS are impacted by the cognitive strain associated with using such avoidance or coping strategies.

In best practice (Ministry of Justice, 2022), police investigative interviewers typically ask witnesses to freely recall events in detail without interruption (‘free recall’), probing with follow‐up questions for further detail (‘cued recall’). Free recall not only protects the integrity of memories but also tends to be more cognitively demanding than cued recall, as it requires witnesses to retrieve episodic memories without external prompts, thereby increasing cognitive load (Boraxbekk et al., 2015; Chevroulet et al., 2021). For instance, it is well established that autistic individuals typically experience difficulty retrieving episodic memories, which is exacerbated in unsupported free recall and generally ameliorated under cued recall (Desaunay et al., 2020; Norris & Maras, 2021). However, two contrasting predictions can be made with regard to the effect of free and cued recall on the testimony of PWS. On the one hand, the demands of free recall may intensify cognitive load for PWS, as they must retrieve memories without having explicit cues while still engaging in speech‐monitoring, resulting in diminished recall quality (in terms of completeness or accuracy, or both). Conversely, cued recall may limit the ability of PWS to engage in coping strategies to avoid stammering, such as word substitution, circumlocution or pausing (Lowe et al., 2017; Menzies et al., 2008), as responses are dictated by specific questions. This may increase anxiety and cause a concomitant increase in cognitive load (Chen & Chang, 2017), to the detriment of recall.

Despite the challenges that PWS may experience when providing accounts, no research has examined how they fare when providing eyewitness testimony, nor how they are perceived by others. To address this gap, a series of three mixed methods studies were co‐produced with UK charity STAMMA to examine: (1) the quality (in terms of accuracy and completeness) of eyewitness accounts provided by PWS compared to a cognitive ability‐matched comparison group with no stammer (Study 1a); (2) how PWS experience and navigate the process of providing eyewitness testimony and how they believe the interview process could better support them (Study 1b); (3) the perceived credibility of PWS (Study 2); and (4) whether the provision of information about stammering improves jurors' perceptions (Study 3).

## STUDY 1

Study 1 adopted a mixed methods approach comprising two parts. Study 1a provided a quantitative comparison of memory performance between PWS and a comparison group during a mock eyewitness interview. Based on previous research suggesting that PWS may experience higher cognitive load when recalling past events, it was hypothesized that PWS would report fewer correct details (i.e. reduced completeness), and make more errors (i.e. increased number of incorrect details) and therefore be less accurate (with accuracy operationalized as correct details/correct details + errors) than a comparison group with no stammer. Analyses also examined whether there were differences between groups within the free‐ and cued‐recall phases separately (without directional hypotheses). Study 1b provided a qualitative exploration of PWS's lived experiences of recalling the event under a mock witness interview in the study. Via open‐ended survey responses and follow‐up semi‐structured interviews, PWS were probed on their views on challenges, what worked well and what could be done differently to better support them during interviews.

### Study 1: Method

#### Design

A mixed methods approach encompassed a quantitative comparison of the completeness and accuracy of participants' eyewitness recall between PWS and a comparison group, followed by a qualitative exploration of PWS witnesses' lived experiences of recalling the event under a mock witness interview.

For the quantitative eyewitness accuracy phase (Study 1a), PWS and comparison (no stammer) participants viewed a video of a mock crime before being interviewed on their memory for it. The independent variable was participant group (PWS vs comparison). The dependent variables were number of correct details (completeness), number of incorrect details (errors) and accuracy (number of correct details divided by the sum of correct and incorrect details).

After providing their eyewitness testimony, in Study 1b, the PWS group were asked to provide qualitative feedback on their experiences of giving evidence via open‐ended survey questions. Additional follow‐up semi‐structured interviews provided a further opportunity for PWS to elaborate on their experiences in a focused yet flexible manner (Trainor & Bundon, 2021). This part of the study was grounded in a critical realist framework, enabling a deep and nuanced exploration of the subjective lived experiences of PWS while considering the broader bio‐psycho‐social factors that influenced these experiences (Jordan et al., 2024).

The study design, hypotheses and planned analyses were pre‐registered on the Open Science Framework (https://osf.io/dbqr5/?view_only=06e499b49446401dae6c86a6a9f614a4).

#### Participants

All participants were required to be a UK resident, 18 years of age or older and proficient in English at a first‐language level. Additionally, PWS had to have a current stammer, irrespective of its duration. Recruitment occurred through purposive sampling (Nyimbili & Nyimbili, 2024), using advertisements on closed social media groups (Facebook, LinkedIn), the University of Bath's Psychology Community Research Panel and STAMMA's research platform.

Eighty‐one participants took part in the study; however, 14 were excluded for either not residing in the United Kingdom (*n* = 10) or failing to replicate their participant‐generated ID code (*n* = 4). This resulted in a final sample of 67 participants: 26 in the PWS group and 41 in the comparison group. Demographics and mean cognitive ability scores are shown in Table 1. An independent samples *t*‐test indicated the PWS group was significantly older than the comparison group, *t*(45.64) = −4.15, *p* < .001, *d* = 1.09, 95% CI [−1.61, −0.56], and a chi‐square test revealed a statistically significant difference in sex distribution between groups, *χ* ^2^(1) = 10.66, *p* = .001, Cramer's *V* = .40, OR = 5.69, 95% CI [1.93, 16.79], with relatively more females in the comparison group. However, there was no difference in cognitive ability score (as measured by the Self‐Administered Vocabulary IQ Test – see Materials section below) between groups, *t*(40.78) = −0.51, *p* = .616, *d* = 0.14, 95% CI [−0.64, 0.37].

**TABLE 1:** Demographics and cognitive ability score of the stammering and comparison group in Study 1.

	Stammering (*n* = 26)	Comparison (*n* = 41)
Age (years)	37.5 (11.3); range: 22–59	26.5 (9.2); range: 18–65
Sex		
Female	10 (38.5%)	32 (78.0%)
Male	16 (61.5%)	9 (22.0%)
Ethnicity		
Asian	4 (15.4%)	19 (46.3%)
White	16 (61.5%)	19 (46.3%)
Black	6 (23.1%)	2 (4.9%)
Prefer not to say	0 (0.0%)	1 (2.4%)
Cognitive ability score	98.96 (15.90)	97.02 (12.94)

*Note*: Standard deviations for age and cognitive ability score are in parentheses.

PWS participants received a £15 Amazon voucher upon completion, while the comparison group (whose participation was shorter as they did not complete the post‐testimony reflections survey/interview) received a £10 voucher. The study was approved by the University of Bath Research Ethics Committee (reference number: 5309‐6811).

#### Materials and procedure

All participants took part individually online. First, participants viewed a 2‐min crime video via screenshare. The film was originally created for use in a previous study (see Maras et al., 2020) and depicted a fight between two males, witnessed from the perspective of a bystander. After viewing the video (and before being interviewed on their memory for it), participants completed demographic and cognitive ability questions on QuestionPro, taking around 15 min.

##### Online self‐administered vocabulary IQ test (SA‐VIQT)

The SA‐VIQT (available at https://openpsychometrics.org/) consists of 45 cognitive ability questions whereby participants select two out of five words that share the same meaning. Participants receive one point for each correct answer, and one point is deducted for each incorrect answer (‘Don't know’ responses yield zero points). The test demonstrated moderate convergent validity, with Pearson's *r* ranging from .48 to .54 (Logos et al., 2021).

##### Eyewitness testimony interview

Next, participants were interviewed on their memory of the video by one of four research assistants (three females and one male, aged between 22 and 24 years). Interviews followed a standardized schedule designed to incorporate best practice for interviewing witnesses (Ministry of Justice, 2022). Initially, participants were asked to recall all details they remembered, but only those they were confident were accurate (‘free recall’). They were then asked a series of questions based on their free recall accounts using orienting ‘tell’, ‘explain’, ‘describe’ prompts (e.g. ‘You mentioned someone tried to intervene; please describe this person in as much detail as you can remember’).

##### Qualitative post‐testimony reflections survey

After the eyewitness interview, participants in the stammering group received a link to a survey asking about their experiences of providing testimony. The survey was developed based on existing empirical findings. In line with participatory research principles, which emphasize the value of co‐producing knowledge through systematic inquiry in direct collaboration with the populations being studied (e.g. Pérez Jolles et al., 2022), the survey (and wider set of studies more broadly) was co‐produced with an individual with lived experience of stammering and supporting other PWS. It comprised 20 open questions and seven closed questions, divided into six sections: memory, ability to articulate details, emotional impact of the interview, management of stammer during the interview, influence of interviewer reactions and suggestions for improvement (Supplementary Material A).

##### Qualitative post‐testimony reflections interview

Following a review after 3 weeks of data collection, it was determined that survey responses lacked sufficient depth. Consequently, an interview schedule was developed to further explore the topics most salient in survey responses. PWS participants who had completed the initial qualitative survey were contacted to participate in a follow‐up interview; subsequent participants were given the option to complete either the survey or follow‐up interview. Ten PWS participants completed this final optional phase. Twelve open‐ended questions covered key topics, but discussions were further guided by what the researcher interpreted to be meaningful to the participant (Trainor & Bundon, 2021). The interview schedule mirrored the survey's focus areas, including memory and articulation of event details, cognitive and emotional impact, interviewer reactions, coping strategies and suggestions for improvement (Supplementary Material B).

#### Coding

##### Eyewitness testimony

The recorded eyewitness interviews were transcribed and coded by the third author. Each new item of detail reported by participants was coded as correct (if it matched that seen in the video) or incorrect (if it did not match, was not present or was recalled as occurring in a different sequential order to what was shown in the video). The accounts of participants were coded at the finest level of detail available. For instance, ‘A female [correct] came over [correct]. She was wearing a blue [incorrect] jumper [correct]’ would receive three correct points and one incorrect point (her jumper was burgundy).

Items were coded only once the first time they were mentioned, but items were coded multiple times if they were necessary for understanding the context. For instance, the male friend was coded multiple times when participant reported ‘The male friend [correct] tried to intervene [correct] but one guy [correct] pushed [correct] the male friend [correct] away’. Items that were subjective (e.g. ‘He looked more aggressive’) were not coded. Twelve transcripts (17.91%) were randomly selected and independently coded by a second researcher. Excellent agreement was reached between the two raters, with intraclass correlation coefficients of >.99 (95% CI [0.996, 0.998]) for correct details and .99 (95% CI [0.98, 0.99]) for incorrect details.

##### Surveys and interviews with PWS

PWS participants' post‐testimony reflections were coded using an inductive reflexive thematic analysis by the second author. Adopting a critical realist perspective, the analysis recognized that participants' interpretations of their experiences were influenced by their broader ideologies, social contexts and prior stammering experiences (Braun & Clarke, 2022). The researcher's interpretations were also recognized as contributing to the construction of the analyses. The analysis was carried out at a semantic level, focusing on the explicit meaning of what participants said (Braun et al., 2023).

Braun and Clarke's (2006) six‐stage approach was flexibly followed, comprising (1) familiarization with the data through reading and re‐reading responses; (2) generating initial codes reflecting potentially interesting data features regarding the research question; (3) generating initial themes by grouping similar codes to elucidate shared meaning; (4) reviewing themes to ensure they tell the story of the full dataset; (5) refining and defining themes; and (6) developing the analytic narrative through contextualizing the themes in existing literature. Throughout, the researcher remained reflexive, iteratively revisiting and refining codes and themes to ensure they remained true to the data and adequately reflected the complexities of participants' experiences. The coding was discussed with another member of the research team, and it was agreed that the codes accurately reflected participants' experiences.

### Study 1: Results

#### Eyewitness testimony

Three independent samples *t*‐tests were conducted to examine group differences in completeness (number of correct details), errors (number of incorrect details) and accuracy (number of correct details divided by the sum of correct and incorrect details), respectively. Due to unequal group sizes, Welch's *t*‐tests were used. These tests were repeated in further analyses to test for group differences in completeness, errors and accuracy within the free recall and cued recall phases separately.

##### Overall recall performance

There were no significant differences between PWS and comparison groups across the interview overall, in terms of the number of correct details, Welch's *t*(58.65) = 1.54, *p* = .129, *d* = 0.37, 95% CI [−0.12, 0.87], errors, *t*(56.68) = 1.69, *p* = .097, *d* = 0.42, 95% CI [−0.83, 0.91] or overall accuracy, *t*(50.25) = −0.90, *p* = .372, *d* = 0.23, 95% CI [−0.72, 0.26] (Table 2).

**TABLE 2:** Mean number of correct details, errors and accuracy for PWS and comparison groups across the interview overall (standard deviations in parentheses).

	Comparison (no stammer)	PWS
Overall interview		
Correct details	75.61 (19.52)	68.65 (17.00)
Errors	10.85 (5.09)	8.81 (4.67)
Accuracy (proportion)	0.87 (0.06)	0.89 (0.06)
Free recall		
Correct details	46.71 (16.21)	38.31 (13.63)
Errors	3.39 (2.31)	2.65 (2.06)
Accuracy (proportion)	0.93 (0.05)	0.93 (0.06)
Cued recall		
Correct details	28.90 (13.42)	30.35 (10.04)
Errors	7.46 (5.02)	6.15 (3.96)
Accuracy (proportion)	0.79 (0.12)	0.83 (0.08)

##### Free recall

In the free recall phase, comparison witnesses reported significantly more correct details than the stammering witnesses, Welch's *t*(59.86) = −2.28, *p* = .026, *d* = 0.55, 95% CI [0.05, −1.05]. There were no differences between groups for errors, *t*(57.86) = 1.36, *p* = .179, *d* = 0.33, 95% CI [−0.16, 0.82] or accuracy, *t*(44.47) = −0.06, *p* = .950, *d* = 0.02, 95% CI [−0.51, 0.48] (Table 2).

##### Cued recall

There were no significant differences between PWS and comparison witnesses in number of correct details, *t*(63.11) = −0.50, *p* = .617, *d* = −0.12, 95% CI [−0.61, 0.37], incorrect details, *t*(61.83) = −1.19, *p* = .240, *d* = 0.28, 95% CI [−0.21, 0.78] or accuracy, *t*(63.69) = 1.85, *p* = .069, *d* = 0.43, 95% CI [−0.93, 0.07] during the cued recall phase (Table 2).

#### PWS reflections on providing testimony

**FIGURE 1:**
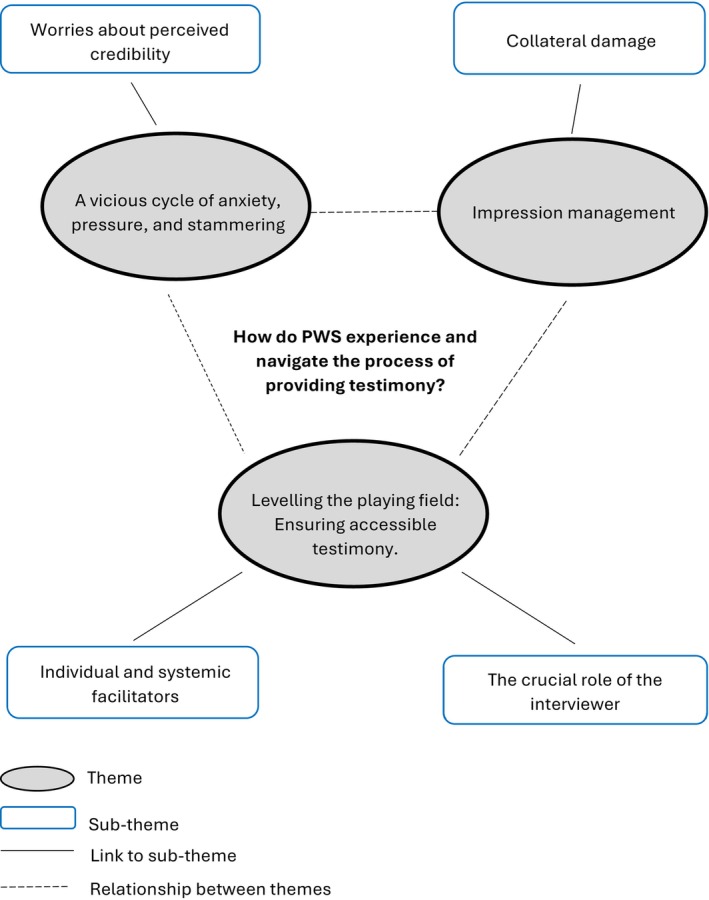
Thematic map illustrating the three themes and four subthemes of participants' reported experiences.

The inductive reflexive thematic analysis of PWS participants' reflections (in semi‐structured interviews and survey responses) of providing testimony generated three overarching themes: (1) ‘A vicious cycle of anxiety, pressure, and stammering’ (subtheme: ‘worries about perceived credibility’); (2) ‘Impression management’ (subtheme: ‘collateral damage’); (3) ‘Levelling the playing field: ensuring accessible testimony’ (subthemes: ‘individual and systemic facilitators’ and ‘the crucial role of the interviewer’). A thematic map is presented in Figure 1, while Supplementary Material C presents each theme and subtheme with their associated codes and example pseudonymized quotations.

#### Theme one: A vicious cycle of anxiety, pressure, and stammering

Participants expressed that the process of providing testimony inherently disadvantages PWS compared to fluent speakers. While participants acknowledged that ‘the stress and pressure alone would affect anyone’ (Matthew, 29, male), they emphasized that having a stammer introduces an additional layer of difficulty. A core element of the anxiety experienced by PWS was their ‘fear of stammering’ (Oliver, 44, male). Participants reported that a desire to avoid stammering contributed to a cycle, whereby the high‐pressure environment heightened their anxiety about stammering, which in turn further increased the frequency and intensity of stammering. Moreover, many participants pointed to the added anxiety of speaking in front of multiple strangers, regarded by Ruby (32, female) as ‘shit scary’. Participants felt that the high‐stakes performance demands of providing testimony inhibited their ability to cope with their stammer.

##### Subtheme: Worries about perceived credibility

For many participants, anxiety over speaking in front of others went beyond typical fears of public speaking; it was deeply rooted in concern over one's articulation abilities and the impact of these on listeners' perceptions. Participants reported acute awareness that ‘[…] for most people in the world stammering isn't something they are familiar with; they don't understand it’ (Oliver, 44, male), which contributed to concerns about how their testimonies might be misinterpreted in the CJS. A common fear amongst participants was that the societal bias linking fluency with credibility would undermine listener's perceptions of their honesty, and they expressed frustration with the lack of understanding about how stammering manifests. For some, the fear of appearing dishonest went beyond a mere point of anxiety to profoundly affect their belief in their reliability as witnesses, driven not by one's ability to recall events but by anticipation of listeners misjudging them as dishonest. For example, Sophia (31, female) remarked that she ‘would feel useless in a real‐life crime investigation’, indicating a dissonance between internal confidence in the accuracy of testimony and the anticipation of negative external perceptions.

#### Theme two: Impression management

As a result of their anxiety over listener misperceptions, many participants experienced a constant internal conflict between trying to speak fluently and accurately articulating their testimony. Participants felt preoccupied with anxiety over how they were being perceived by listeners, ‘Like if we do stammer, what impact that will have on listeners and also us, in terms of like … you know … our confidence for example, or like how we perceive the listener to be viewing us’ (Ben, 24, male). Participants indicated that providing testimony as a PWS is entangled with fears of social stigma, leading them to be hypervigilant for listeners' reactions and focusing less on testimony content and more on navigating ‘[…] what can go wrong with how they're perceived’ (Louie, 49, male).

Several participants attempted to manage listener misperceptions by employing pre‐emptive avoidance strategies to maintain fluency, including avoiding certain words or sounds that they knew would induce their stammer. For example, Ruby (32 female) noted ‘I also try to use alternate words most of the time when I am unable to speak a particular word. But I have also realised that the essence of the story is lost most of the times when using alternate words’.

Many participants also reported limiting their responses to avoid stammering or managing their stammer by breaking eye contact to regain control of their speech: ‘I mean it's better to not speak than to sort of get myself into more problems’ (Louie, 49, male).

##### Subtheme: Collateral damage

The pre‐emptive employment of avoidance strategies fed into a trade‐off between participants' prioritization of fluency over testimony content. Several participants explicitly expressed fears over the impact of this trade‐off on the clarity of their testimony. Most felt that although they were able to convey their intended message, the use of avoidance strategies made their testimony less precise and clear and thus compromised its effectiveness. For many participants, the effort it took ‘to go around the houses’ (William, 45, male) was ‘exhaustive’ (Ruby, 32, female) and ‘draining’ (William, 45, male). This cognitive burden comprised a complex interplay between fears of listeners' perceptions, employment of avoidance strategies and testimony articulation.

Contrastingly, a few participants prioritized the accuracy of their testimony over appearing fluent to listeners. For these participants, testimony content closely aligned with what they intended to say because, as Megan (40, female) explained, ‘Even if I knew I would stammer it was important to say what I wanted to say’.

Despite variability in stammer management, participants consistently asserted that their memory of events remained unaffected, with Talia (47, female) noting that she could ‘recall the whole incident vividly’. Instead, several participants acknowledged that the difficulty lay in effectively communicating these memories; ‘the way I talk is the only thing that is affected here’ (Jacob, 30, male).

#### Theme three: Levelling the playing field – Ensuring accessible testimony

Participants emphasized the need for accommodations to ensure that the testimony process is accessible and equitable for PWS. Drawing parallels with physical disabilities, many argued that the same level of consideration must be given to PWS, as conveyed by Kaya's (32, female) remark that ‘[…] if you had a courtroom and you had somebody in a wheelchair, you wouldn't ask them to walk up a staircase’.

Participants felt that the right accommodations would enable PWS to provide effective testimony, with Matthew (29, male) stating, ‘So, make the allowances and I don't see any reason why we couldn't’. However, participants stressed that these accommodations must consider the unique needs and abilities of each individual because ‘it's not a one size fits all’ (Matthew, 29, male).

##### Subtheme: Individual and systemic facilitators

Participants identified a range of strategies and adjustments to facilitate the testimony process and overcome the challenges outlined previously. These facilitators consisted of both individual coping strategies (e.g. slowing speech, taking time and rehearsing beforehand) and systemic accommodations (e.g. familiarization with processes). For example, Logan (59, male) notes: ‘I would want to meet the judge in person and have a look round the venue and familiarise myself with the court and the judge’.

Of particular importance to participants was the need to disclose their stammer to set listener expectations and relieve the pressure to remain fluent: ‘If they're waiting … if they're waiting for pauses and things on difficult questions … and they misconstrue the pause as an overthinking or trying to think of a way out of the answer rather than a block … yeah … then perhaps they should be aware that there's a stammer there’ (Louie, 49, male).

Moreover, participants highlighted the need for education to mitigate the impact of prejudices on listener perceptions. Listeners' awareness of ‘their own potential prejudices and stereotypes’ (Logan, 59, male) was also deemed crucial to fostering an environment where PWS feel confident that their testimony will be judged on its content rather than their speech. Further, several participants suggested that offering PWS the option to provide their testimony through alternative mediums, such as in written format, via video link or in a one‐to‐one exchange would further relieve the pressure to appear fluent (while acknowledging this would need to be the individual's choice). As Oliver (44, male) suggests, ‘[…] or possibly have it pre‐recorded so you're not as under so much pressure within a courtroom or a police station or something like that’.

##### Subtheme: The crucial role of the interviewer

Participants expressed the importance of the interviewer‐interviewee dynamic in facilitating – or inhibiting –testimony quality. Benedict (40, male) notes: ‘Because the interviewer was patient, conscientious, and understanding of my speech, it put me at ease, so I was able to talk more fluently’. Participants stressed the importance of establishing familiarity and rapport with the interviewer to reduce their anxiety and facilitate their testimony articulation: ‘I had the opportunity to engage with yourself a bit before, I sort of, you know, had trust in yourself’ (Matthew, 29, male). Equally, participants pointed to the detrimental effect that interviewer impatience, lack of understanding and negative demeanour would have on exacerbating their stammering, and in turn, inhibiting their ability to provide testimony. As Benedict (40, male) notes: ‘If I sense any negativity towards my speech, it makes it worse’.

### Study 1: Discussion

This is the first study to explore eyewitness testimony by PWS. Contrary to hypotheses, PWS and comparison groups did not significantly differ in the completeness, errors or accuracy of their overall accounts, indicating that PWS were able to effectively allocate their cognitive resources to recall events, despite experiencing other demands such as speech monitoring and impression management. However, analyses within the free‐ and cued‐recall interview phases separately indicated that the PWS group reported fewer correct details than the comparison witnesses during free (but not cued) recall. In free recall, witnesses were asked to recall as many details as possible about the crime scene without any explicit prompts on what details should be retrieved. This lack of retrieval support may place excess burden on an already heavy cognitive load for the PWS group, resulting in less detailed eyewitness accounts relative to those with no stammer. These differences were diminished, however, with structured cued recall, which places less demand on cognitive resources (see Glenberg, 1997; Tulving, 1985).

In line with these quantitative findings regarding recall quality, in follow‐up surveys and interviews, the PWS group reported experiencing a cyclical interplay between communicative pressure, anxiety over listener misperceptions and stammer severity, which they navigated by prioritizing either fluency or testimony content. A primary source of reported anxiety was concern over being incorrectly perceived as dishonest or lacking credibility. For some participants, anxiety about external judgements diminished their confidence in their ability to provide testimony. Participants responded to fears over listener misperceptions in two primary ways. Some PWS attempted to avoid stammering through concentrated efforts, aligning with existing literature indicating that PWS often conceal their overt stammer to manage listener reactions (Constantino et al., 2022; Grisham et al., 2015; Jackson et al., 2015). In line with previous research, participants reported using coping strategies such as word‐switching, limiting speech output and avoiding eye contact (Connery et al., 2020; Constantino et al., 2022). However, participants expressed concerns that this compromised the clarity of their testimony. Conversely, other participants prioritized testimony content and chose to speak through their stammer. This variability in experiences highlights the diverse ways that PWS navigate the testimony process and manage their speech during testimony (Garcia‐Barrera & Davidow, 2015; Tichenor et al., 2022). These findings underscore the need to examine how PWS are perceived by others when providing evidence.

## STUDY 2

When considering witness credibility, jurors often (erroneously) rely on perceived behavioural indicators of veracity and truthfulness to inform their judgements (Vrij & Turgeon, 2018). Witnesses are typically also judged on their ability to testify (Cuddy et al., 2009; Neal et al., 2012), how likeable they appear (e.g. how friendly, respectful, well mannered and pleasant) and how confident or certain a witness is regarding the accuracy of their testimony (McClure et al., 2013; Wixted et al., 2015).

Various elements of speech, including fluency, tone, pacing and emotional expression, can shape a jury's perception of the witness (Cramer et al., 2009; Iudici et al., 2023). For example, witnesses who speak ‘powerlessly’, with pauses, hesitations and filler words, are often rated as less credible than witnesses who speak ‘powerfully’ and fluently (Gojkovich et al., 2019; Ruva & Bryant, 2004). Although no research to date has examined juror perceptions of PWS specifically, research indicates that other groups with speech and communication differences, such as individuals with developmental language disorder (Hobson et al., 2022), intellectual disability (Henry et al., 2011) and autism (Lim et al., 2022; but see Maras et al., 2019), are often rated as less credible, even when they are accurately recalling events.

The current study examined the perceived credibility of PWS. Perceived speech quality was also assessed to determine whether credibility perceptions are associated with perceived differences in the fluency and quality of their speech. Based on previous research with other populations, it was predicted that PWS would be perceived as less credible by mock jurors than witnesses who do not stammer. We also predicted that PWS would be scored lower on speech quality than witnesses who do not stammer. After the scale ratings, mock jurors were asked open‐ended explanatory follow‐up questions to contextualize their responses, including whether/how their perceptions changed after being provided with information about stammering.

### Study 2: Method

#### Design

One‐hundred‐and‐fifty mock juror participants were randomly allocated to view and rate one of 30 videos from Study 1 of either a PWS (*n* = 15) or a non‐stammering witness (*n* = 15). Half (*n* = 75) of jurors viewed a PWS, and the other half (*n* = 75) viewed a non‐stammering witness (between participants). This resulted in each of the 30 videos being viewed by five different jurors. The dependent variables were the four subscales of perceived credibility and seven subscales of speech quality. Additionally, mock jurors provided free text responses explaining their reasoning for the credibility and speech scale ratings, which were subject to an inductive content analysis.

The study design, hypotheses and planned analyses were pre‐registered on the Open Science Framework (https://osf.io/wfb2z?view_only=2640b4aaf6da480c9de1a6803523f0a5).

#### Participants

An a priori power analysis using G*Power version 3.1.9.7 (Faul et al., 2007) indicated that a total sample size of 126 participants would give 80% power to detect a medium‐to‐large effect size (Cohen, 1988). One hundred and fifty mock juror participants took part via QuestionPro or subsequently (due to a technical issue) Qualtrics. They were recruited through Prolific.com using convenience sampling and were paid according to Prolific's payment principles (approximately £4.50). To be eligible for the study, participants were required to be age 18 or over, able to read, write and understand English as a first language or equivalent, and reside in the United Kingdom. Due to an oversight in survey development, demographic information was not collected.

Ethical approval was obtained from the University of Bath Social Science Research Ethics Committee (reference number: 5309‐5733).

#### Materials and procedure

##### Eyewitness testimony videos

A total of 30 videos (15 PWS and 15 comparison witnesses) were selected from the wider Study 1 sample of 26 PWS and 41 comparison witnesses. Multiple interview videos were used to capture a diverse range of stammers, while trimming witness groups from the wider Study 1 sample to ensure they were matched more closely on age, gender and ethnicity.^1^ All 30 witnesses gave consent for their videos to be used in the current study. The videos were edited to contain only the free recall phase of the interview (which lasted between 2 and 5 min).

Mock juror participants were randomly allocated to watch a video of either a PWS or non‐stammering witness. They were instructed to watch the entire video and have the page set to full‐screen mode.

##### Witness credibility scale

Perceived credibility was assessed using a 20‐item adapted version of the ‘Witness Credibility Scale’ (Brodsky et al., 2010). Each item contains bipolar adjectives that are rated on a 10‐point Likert scale (e.g. unreliable [1] to reliable [10]). A score of 1 indicates a strong agreement with the negative attribute while a score of 10 indicates a strong agreement with the positive attribute. The scale measures credibility across four subscales (Likeability, Trustworthiness, Confidence and Knowledge), each made up of five items. As the scale was originally developed for expert witnesses, four items were modified and the Knowledge subscale was renamed Capability (see Supplementary Material D). Subscale scores (out of 10) are calculated by averaging the ratings of the five corresponding items. The Witness Credibility Scale has good reliability (*α* = .95), and the four subscales yield consistently high overall internal consistency in credibility scores (.91–.98; Brodsky et al., 2010).

Two attention check questions were included in the Witness Credibility Scale, asking participants to select a specific Likert score and submit a specified word for the free‐text follow‐up. If participants failed to submit the correct score or word, they were removed from the dataset.

##### Speech quality

The ‘Public Speaking Scale’ (Iberri‐Shea, 2017) is an 11‐item measure that asks respondents to rate various indicators of an individual's public speaking ability (e.g. projection). Items are rated on a 5‐point Likert scale (1 = least favourable evaluation of the speaker; 5 = most favourable). For each item, descriptions of the five points are provided to help guide the rater's decision. Four items were removed from the scale (Introduction, Body, Conclusion and Topic Choice) as they were not relevant to the aims of the current study (see Supplementary Material E). An overall averaged score between 1 and 5 could be obtained, with 5 indicating the highest speech quality. The Public Speaking Scale demonstrates high inter‐rater reliability for measuring overall public speaking ability (*α* = .93), with high inter‐rater reliability for the individual indicators (.87 to 1.0; Iberri‐Shea, 2017).

After completing the scales, jurors in the PWS condition answered open‐ended free text questions probing whether they noticed anything about the witnesses' speech or communication style. They were then informed that the witness had a stammer and were provided information about stammering, taken from STAMMA's website. This included a definition, common characteristics and strategies that PWS often use to mask their stammering (see Supplementary Material F). After reading the information about stammering, participants were asked a final free text question about whether/how this information changed their perception of the witness.

### Study 2: Results

#### Perceived credibility

To examine differences between groups in perceived credibility scores, a one‐way MANOVA was conducted, with type of video shown (PWS vs no stammer) as the independent variable and the composite juror scores for confidence, likeability, trustworthiness and capability as dependent variables. Table 3 displays the mean scores for each of the four perceived credibility outcome variables.

**TABLE 3:** Mean credibility and speech quality scores within each witness video condition (standard deviations in parentheses).

	No stammer	PWS
Credibility		
Confidence	6.78 (1.60)	6.25 (1.69)
Likeability	7.90 (1.05)	8.21 (1.09)
Capability	7.01 (1.58)	7.11 (1.50)
Trustworthiness	7.30 (1.32)	7.62 (1.51)
Speech quality		
Projection	3.76 (1.10)	3.77 (1.02)
Pace	3.60 (1.04)	3.36 (1.18)
Intonation	3.40 (1.15)	3.25 (1.12)
Diction	3.97 (1.17)	3.69 (1.25)
Language use	3.71 (1.12)	3.63 (1.06)
Vocabulary	4.24 (1.08)	4.39 (0.90)
Persuasiveness	3.36 (1.25)	3.41 (1.15)

There was a significant multivariate effect of type of witness video viewed on the combined confidence, likeability, capability and trustworthiness scores, *F*(4, 145) = 4.39, *p* = .002, *ηp* ^2^ = 0.11. However, separate univariate tests on the outcome variables revealed no significant effects of witness group on confidence, *F*(1, 148) = 3.81, *p* = .053, *ηp* ^2^ = 0.03, likeability, *F*(1, 148) = 3.02, *p* = .084, *ηp* ^2^ = 0.02, capability, *F*(1, 148) = 0.14, *p* = .711, *ηp* ^2^ = 0.01 or trustworthiness, *F*(1, 148) = 1.94, *p* = .165, *ηp* ^2^ = 0.01. Although not pre‐registered, we also modelled each measure of perceived credibility using a linear mixed‐effects analysis to account for variation across videos. These models included a fixed effect for type of video (coded PWS = 0.5, no stammer = −0.5) and a random intercept for video. Models were fit in R (R Core Team, 2024, Version 4.3.3) using the lme4 package (Bates et al., 2015, Version 1.1–35.5), and degrees of freedom were approximated using Satterthwaite's method via the lmerTest package (Kuznetsova et al., 2017, Version 3.1.3). These analyses led to the same conclusions for confidence, *B* = −0.53, *t*(28) = −1.57, *p* = .129, likeability, *B* = 0.30, *t*(28) = 1.72, *p* = .096, capability, *B* = 0.09, *t*(28) = 0.32, *p* = .755 and trustworthiness, *B* = 0.33, *t*(148) = 1.39, *p* = .165.

The MANOVA was followed up with discriminant analysis, which revealed one discriminant function. This explained 100% of the variance, canonical *R* ^2^ = .11. This discriminant function significantly differentiated the type of witness video viewed between groups, Λ = .89, *c* ^2^(4) = 16.70, *p* = .002. The correlations between outcomes and the discriminant function revealed that likeability (*r* = .41) and trustworthiness (*r* = .33) both loaded moderately onto the function. Capability loaded weakly onto the function (*r* = .09), while confidence loaded moderately but negatively onto the function (*r* = −.46). This indicates that the witness groups were distinguished by a variate that has opposite effects on different dimensions of credibility. Considering the means from Table 3 and the correlations between outcomes and the discriminant function, it appears that PWS were viewed as less confident but more likeable and trustworthy. Perceptions of capability were similar between witness groups.

#### Speech quality

To examine differences between groups in perceived speech quality, a second one‐way MANOVA was conducted. Type of video shown (PWS vs no stammer) was the independent variable, and the juror scores for projection, pace, intonation, diction, language use, vocabulary and purpose were dependent variables. There was no significant multivariate effect of witness video viewed on speech quality, *F*(7, 142) = 1.54, *p* = .158, *ηp* ^2^ = 0.07 (Table 3).

#### Relationship between overall speech quality and perceived credibility

A simple linear regression analysis found that overall speech quality was positively associated with higher ratings of overall witness credibility, *B* = 0.74, *β* = .54, *t*(148) = 7.80, *p* < .001, *r* ^2^ = .29. A linear mixed‐effects model predicting perceived credibility from overall speech quality, with a random intercept for video, revealed the same conclusion: *B* = 0.77, *t*(142.89) = 7.90, *p* < .001. We also tested a model that included a random slope by video for the relationship between credibility and speech quality, but it was flagged as a singular fit, indicating that the random slope variance was near zero and did not meaningfully vary across videos.

#### Content analysis of responses to free‐text questions

The free‐text responses to the final open‐ended questions from jurors who viewed a PWS video were analysed by the first and fifth authors using an inductive qualitative content analysis, following Mayring's (2015) guidelines.

##### Speech distinctions

When reflecting on whether they noticed anything about the witness's speech or communication style (prior to being informed the witness had a stammer), jurors frequently mentioned the stammer itself (with some referring to it as ‘delayed’ or ‘awkward’). Few mentions were made of other speech aspects, such as nervousness and pauses (see Table 4).

**TABLE 4:** Code frequencies and example quotes for the open‐ended question on noticeable speech distinctions in PWS group.

Code	*N* mentions by jurors	Example quotes
Stammer	36	‘Yes, the witness had a stutter. He seemed a bit embarrassed about his stutter and frequently sped up or changed his tone to avoid the awkward experience of pausing’
Accent	5	‘He had an accent and from the use of words, looks like English is not his first language’
Nervousness	6	‘He did stammer or stutter a little but, it didn't affect his being understood & just came across as if he was a little nervous, which is understandable in the situation’
Pauses	4	‘I noticed he was slow and had to take pauses at intervals before he could say words’
Other (shaky, delayed speech, broken English, awkward)	9	‘It was quite slow and it was not always in complete sentences’
Nothing	16	‘Nothing at all’

##### Effect of receiving information about stammering

Finally, analysis of jurors' free text responses after they had received knowledge about the witness's stammer indicated they felt that this information generally improved their credibility perceptions of the witness. Specifically, jurors indicated that they realized that the stammering was not due to nervousness or a lack of credibility but a condition that the witness was struggling with. Jurors also reported becoming more empathetic and understanding of the witness's mental and emotional state due to their stammer (see Table 5).

**TABLE 5:** Code frequencies and example quotes for the open‐ended question on effect of receiving stammer information on perceptions of PWS.

Code	*N* mentions by jurors in stammer condition	Example quotes
Still believes account	12	‘No, people with a stammer are just as reliable for providing witness statements. It may mean the witness delivers his account more slowly if experiencing stammers, but this does not take away from the overall quality’
Rules out nervousness	6	‘Slightly. As said previously. I tell if the stutter was due to him having a genuine stutter or if he was nervous and had something to hide’
Information improves credibility	14	‘At some points I thought the witness was hesitating in their account because they weren't sure of what they saw. Knowing that they have a stammer means I trust their account more’
More empathy for witness	3	‘Yeah in a way, it makes you think about the emotional affect his stammer could have on him and how it maybe deters him from adding lots of details as he might struggle to get it all out’

### Study 2: Discussion

Study 2 examined the perceived credibility and speech quality of PWS and witnesses with no stammer. Mock juror ratings of witness credibility (measured on confidence, likeability, trustworthiness and capability) were significantly affected by the type of witness video they viewed; however, not as initially expected. PWS were perceived as less confident, but more likeable and trustworthy than witnesses who do not stammer, while perceived capability did not differ between groups. The difference in perceived confidence does, however, mirror Study 1 findings, whereby PWS reflected how concerns about others' perceptions of them diminished their confidence in their own ability to provide testimony. This is consistent with the notion that anticipated negative evaluation can lead to internalized self‐stigma in PWS (Fitzgerald et al., 2021; Nang et al., 2018).

Speech quality was positively associated with higher ratings of overall perceived credibility but, perhaps critically, mock juror ratings of speech quality did not significantly differ between PWS and comparison witnesses, which may explain the inconsistent findings regarding credibility. Nevertheless, juror participants' responses to the open‐ended follow‐up questions indicated that they did notice witnesses' stammers, but that formal disclosure of the stammering would have improved their credibility ratings of them.

## STUDY 3

Study 3 aimed to experimentally confirm findings from the qualitative analysis in Study 2, which indicated that providing information about stammering would improve credibility ratings of PWS.

### Study 3: Method

#### Design

Utilizing a between‐subjects design, mock juror participants viewed a video of a witness with a stammer and were randomly assigned to one of two conditions: (i) ‘information’, in which they were informed that the witness had a stammer and were provided information about stammering; or (ii) ‘no information’, in which they received no information about stammering. Dependent variables were mock jurors' ratings of perceived credibility across four subscales: confidence, likeability, trustworthiness and capability. We also included a measure of participants' knowledge of stammering to confirm that the provision of information about stammering improved knowledge about the condition in the first instance.

#### Participants

An a priori power analysis using G*Power 3.1 (Faul et al., 2007) indicated that to detect a medium‐large effect size (based on Hobson et al., 2022; Maras et al., 2018), a minimum sample size of 126 was necessary to achieve Cohen's (1988) recommended power of .80. An opportunity sample of 162 mock juror participants eligible for UK jury service (18–75 years old and UK citizen) was recruited to take part. Recruitment took place through advertisement on messaging and social media platforms, as well as through the University of Bath's Research Participation Scheme. Data from seven participants were subsequently excluded for failure to select the correct response on an attention check question, resulting in a final sample of 155. There was no difference between experimental groups in terms of age, *t*(151) = 0.71, *p* = .225, *d* = 0.20, or gender distribution, Fisher's exact test, *p* = .572 (Table 6).

**TABLE 6:** Mock juror demographics for Study 3.

	No information (*n* = 76)	Information (*n* = 79)
Mean age (SD)	39.11 years (15.78); range = 18–67	36.00 years (15.72); range = 18–64
Gender		
Female	46	51
Male	26	27
Other	1	0
Prefer not to say	3	1

Ethical approval was granted by the University of Bath's Psychology Research Ethics Committee (reference number: 3928‐3947).

#### Materials and procedure

Mock juror participants completed the study online via QuestionPro. After completing the screening and demographic questions, they were randomly assigned to either the information condition or the no information condition.

##### Information about stammering

Participants in the information condition were informed that the witness video they were about to view was of a PWS, and they were provided information about stammering (see Study 2 and Supplementary Material F for details).

##### Eyewitness testimony

Next, mock juror participants read the written transcript of the free recall component of a male witness' testimony before viewing a video of him being questioned by an advocate. To create the eyewitness testimony video, a 73‐year‐old white male volunteer with a stammer was recruited from STAMMA. He viewed a video of a staged theft lasting 1 min 36 s before, 2 h later, completing a written account of what he witnessed in the video. His written account was then shared with an experienced legal advocate who prepared a line of questions with which to test his account. Four days after watching the video, the witness was questioned by the advocate via video call during which he was first asked to provide a verbal free recall of the events before being cross‐examined on this account. The free recall component was transcribed, and the cross‐examination was presented as a 5‐min video.

##### Witness credibility scale

Mock jurors then rated the witness' credibility using the Witness Credibility Scale (Brodsky et al., 2010). See Study 2 and Supplementary Material D for details.

##### Knowledge of stammering questionnaire

Finally, jurors completed a knowledge of stammering measure, adapted from Campbell and Barger's (2010) ‘Knowledge of Autism Questionnaire’. Statements such as, ‘Stammering can involve someone repeating sounds or words’, were developed in collaboration with STAMMA (see Supplementary Material G). Participants responded ‘True’, ‘False’ or ‘Unsure’ (an option added to avoid forced responses). Cronbach's alpha indicated acceptable reliability (*α* = .65).

### Study 3: Results

#### Knowledge of stammering

An independent‐samples *t*‐test examined whether the provision of information about stammering improved participants' knowledge of the condition. Participants who received information about stammering scored significantly higher on the knowledge of stammering questionnaire (M = 9.82, SD = 2.44) than those who did not receive information about stammering (M = 8.83, SD = 2.46), *t*(149) = 2.21, *p* = .014, *d* = 0.36.

#### Provision of stammering information on credibility ratings

To examine if perceptions of credibility differed between the stammering information condition and the no information condition, a one‐way MANOVA was run with the four credibility variables: confidence, likeability, trustworthiness and capability. There was a significant multivariate effect of information provision on perceived credibility, *F*(4, 150) = 3.02, *p* = .020, *h* ^2^_p_ = 0.07. Univariate tests indicated that juror participants who were informed that the witness had a stammer and were given information about stammering rated him as more likeable, *F*(1, 153) = 10.04, *p* = .002, *h* ^2^_p_ = 0.06, trustworthy, *F*(1, 153) = 10.69, *p* = .001, *h* ^2^_p_ = 0.07 and capable, *F*(1, 153) = 4.44, *p* = .037, *h* ^2^_p_ = 0.03 than participants who were not informed about his stammer. There was no significant difference between conditions in terms of perceptions of his confidence, *F*(1, 153) = 4.45, *p* = .063, *h* ^2^_p_ = 0.02 (Table 7).

**TABLE 7:** Mean credibility ratings by information condition (standard deviations are in parentheses).

	No information about stammering	Information about stammering
Confidence	4.34 (1.15)	4.68 (1.10)
Likeability	5.33 (0.79)	5.73 (0.75)
Trustworthiness	4.55 (1.06)	5.11 (1.06)
Capability	4.43 (0.99)	4.77 (1.01)

### Study 3: Discussion

As predicted, jurors who were informed that the witness had a stammer and were provided with information about stammering rated him as more credible overall, and more likeable, trustworthy and capable specifically. However, perceptions of the witness's confidence were unaffected by information provision.

## GENERAL DISCUSSION

In this first empirical examination of eyewitness testimony and stammering, we examined the evidential quality (Study 1a), lived experience (Study 1b) and perceived credibility (Studies 2 and 3) of witnesses who stammer. Across three studies, four key findings emerged. First, while PWS recalled a similar amount of information (and as accurately) as cognitive‐ability matched comparison participants across the interviews overall, they recalled fewer correct details during the initial free recall phase of the interview. This difference between groups dissipated in the cued recall phase. Second, PWS reported experiencing the interview as a cyclical interplay between communicative pressure, anxiety over how they would be perceived and their stammer severity. They attempted to navigate this by prioritizing either fluency (at the expense of testimony content) or content (often speaking through their stammer to relay their message). Third, in line with these lived experiences, PWS were perceived by mock jurors as less confident, but equally as capable and more likeable and trustworthy than comparison witnesses. Fourth, informing jurors that a witness has a stammer, and providing them with information about stammering, further increased ratings of their likeability, trustworthiness and capability, but had no effect on how confident the witness appeared.

It is positive that, despite the increased cognitive load that PWS reported experiencing due to anxiety, speech monitoring and concerns over others' perceptions, their overall eyewitness recall was as complete and accurate as witnesses who do not stammer. However, the fact that PWS recalled less information in the initial free recall phase of the interview suggests that the additional cognitive pressures of providing an account without external scaffolding diminished the amount of detail that they were willing or able to report (Boraxbekk et al., 2015; Chevroulet et al., 2021). In contrast, and in line with findings with other groups with communication difficulties such as autism (see Norris et al., 2020; Norris & Maras, 2021), there was no difference between groups during cued recall.

Consistent with this experimental finding, follow‐up surveys and interviews with PWS indicated that they navigated the challenges and demands of providing an account by either engaging in avoidance strategies (e.g. circumventing particular words or phrases they know they would find difficult to verbalize without stammering), or prioritizing testimony content and speaking through their stammer. Notably, individuals who employed avoidance strategies feared this hindered the clarity of their testimony, reflecting the cognitive struggle between achieving fluency and conveying intended messages (Bricker‐Katz et al., 2013; Iimura et al., 2016), paralleling the experiences of bilingual speakers providing testimony in their non‐native language (Akehurst et al., 2018; Alm et al., 2019; Hu & Naka, 2023).

An interesting avenue for future research will be to examine eyewitness recall by PWS in the context of Koriat and Goldsmith's (1996) monitoring and control framework, and in particular their strategic regulation of grain size in memory reporting (Goldsmith et al., 2002; Weber & Brewer, 2008). Within this framework, individuals balance competing goals of accuracy and informativeness by making a series of reporting decisions, such as whether to volunteer a detail (and if so, at what level of granularity) or withhold the detail altogether (Goldsmith et al., 2002). In Study 1, PWS reported needing to prioritize either fluency or accuracy, suggesting that the additional cognitive burden associated with providing an account without external cues resulted in fewer correct details reported in the free recall phase. However, based on the framework of Koriat and Goldsmith (1996), it is possible that PWS simply adopted a more cautious reporting strategy by raising their reporting threshold, choosing in their free recall to communicate only details they were highly confident in. Future studies should incorporate a confidence measure to examine the confidence–accuracy relation at the statement level.

Given that PWS appear to find unstructured recall more challenging, adapted interview techniques for witnesses who stammer may be appropriate. For instance, Maras et al. (2020) developed a Witness‐Aimed First Account (WAFA) interviewing technique for autistic witnesses – a population who experience difficulties in recalling episodic events in free recall. Unlike typical free recall, WAFA encourages individuals to self‐segment the event they witnessed, which is later displayed on Post‐It notes or similar as a reminder of the structure of the event (Maras et al., 2020). WAFA has been shown to yield higher accuracy and completeness of eyewitness accounts by both autistic and non‐autistic adults, and may be a fruitful line of investigation for PWS.

Stammering appeared to influence mock juror perceptions of witness credibility in a more heterogenous manner than anticipated. Specifically, PWS were rated as more likeable and trustworthy, equally as capable, but less confident than comparison witnesses. Whereas previous research found that other groups with communication difficulties (e.g. developmental language disorder, intellectual disability and autism) are often perceived more negatively across credibility domains (Henry et al., 2011; Lim et al., 2022; Spaulding & Blewitt, 2024; but see Maras et al., 2019), stammering seems to differentially influence aspects of witness credibility. This somewhat counterintuitive finding that reduced perceived confidence of PWS was accompanied by increased trustworthiness and likeability challenges the conventional view in forensic psychology that confidence is an important positive determinant of perceived witness credibility (e.g. Slane & Dodson, 2022). It does align, however, with Andrus' (2012) theoretical framework, which posits that the legal system constructs credibility through the appearance of diminished discursive agency. Andrus outlines how spontaneous, emotionally unfiltered, less polished speech is often treated as more trustworthy because of its lack of artifice: The speaker is perceived to be unable to fabricate a story, and their account appears more authentic. Accordingly, it may be the case that PWS are seen as trustworthy primarily due to perceptions of diminished discursive agency.

This has implications for the language ideology embedded within UK and US law (such as Res Gestae evidence and the excited utterance exception to the hearsay law), which not only determines which kinds of speech are admissible in court but also shapes who is seen as a credible speaker (see Andrus, 2012; Coffey, 2023). There is a general tendency of the justice system to be unable to co‐consider agency and vulnerability; discursive agency and trustworthiness are seen as mutually exclusive. That is, a speaker is either agentic and reflective and thus perceived as less trustworthy, or emotional and impulsive and thus seen as more trustworthy. By legally privileging utterances made in emotional states, the system effectively credits speakers when they are presumed to not be in control of what they say (i.e. their statements were an excited utterance). This tension highlights the limited binary structure embedded in the legal system (Andrus, 2012), raising questions around why concepts such as confidence are viewed as essential to credibility, and why this might not always be accurate or serve all populations equally (see also Brown et al., 2017).

It may be pertinent that stammering is a more readily observable characteristic to an outside observer than that associated with other disorders such as autism or developmental language disorder. Indeed, many participants in Study 2 commented on witnesses' stammers. As such, PWS may be perceived as vulnerable from the outset, and likeable and trustworthy as a result. This is in line with research showing that individuals perceived as vulnerable are usually viewed as being higher in warmth (Fiske et al., 2007). The perception of vulnerability may also evoke empathy towards witnesses who stammer due to their noticeable struggle with speech, which jurors might have equated with honesty and reliability (Green & Brock, 2000). Although this has implications for witnesses whose stammering is more covert (i.e. through the deployment of masking, copying or avoidance strategies) and who therefore appear fluent, even though they may not be recalling everything that they would otherwise wish to. Equally, witnesses' stammers – and the para‐ and non‐verbal behaviours associated with managing them – appear to negatively influence juror perceptions of their confidence. This finding aligns with previous research suggesting that nervous and tense body language negatively affects juror perceptions of witnesses' confidence (Iudici et al., 2023; Wessel et al., 2006) and that stammering is a sign of nervousness or lack of confidence (Amick et al., 2017; Boyle, 2017; Sibanda & Mothapo, 2024).

The finding that information provision increased juror credibility ratings of testimony in Study 3 is consistent with previous research with other groups with communication disorders such as developmental language disorder (Hobson et al., 2022; Horsham et al., 2025) and autism (Crane et al., 2018; Maras et al., 2018, 2019). They are also consistent with Amick et al. (2017), who found that increased familiarity with stammering led to reduced social stigma and more positive social perceptions of people who stammer. A likely explanation is that improving knowledge of stammering enables jurors to attribute atypical behaviours – both stereotypical ones (e.g. hesitations and elongations of syllables) as well as lesser known characteristics (e.g. facial grimacing, rapid blinking and strategically navigating syntax) – to the condition, rather than as indicative of a less competent and credible witness (see Kelley, 1973). However, jurors appear to be over‐compensating for the stammer in their decision‐making process, given that in Study 2 (where jurors were not provided with prior information about stammering) PWS witnesses were already rated as more likeable and trustworthy than non‐stammering witnesses; a difference that was amplified with information provision in Study 3. Similar findings have been reported for autistic witnesses, whereby the provision of autism diagnosis information for jurors resulted in them rating autistic witnesses as more credible than their non‐autistic comparisons, despite no differences in the completeness or accuracy of testimony per se (Maras et al., 2019). It may be pertinent, however, that in Study 2, there was no difference between PWS and comparison witnesses in ratings of their speech quality, yet speech quality predicted credibility. It is possible that the free recall component (which was shown to mock jurors) enabled PWS to employ successful masking strategies (albeit at the expense of the completeness of their accounts), resulting in them being perceived as more credible than they would otherwise.

The only aspect of credibility that the provision of information did not affect was perceived witness' confidence. This might be due to ingrained societal biases that equate fluency and smooth speech with confidence (Guyer et al., 2021), or it might simply reflect a genuine lack of confidence on the part of the witness – a supposition supported by the qualitative follow‐up survey and interviews with PWS in Study 1b. It is worth noting, however, that jurors rated how confident the witness appeared generally, rather than how confident they appeared specifically in terms of the reliability of their account. This is an important point for future research to address, particularly in light of previous evidence that fears over misperceptions create a dissonance between PWS's internal confidence in their ability to provide testimony and the anticipated external misperceptions of listeners (Boyle, 2018).

### Practical implications

Findings have several practical implications, not least in highlighting the need for individual and systemic accommodations for PWS in providing testimony. Participants expressed confidence that the right adjustments could mitigate the negative impact of testimony contexts, facilitating the clarity and accuracy of their testimony, but that this requires a multifaceted approach. In their post‐testimony reflections, PWS discussed the importance of individual accommodations, such as providing extra time, incorporating pauses and allowing appropriate preparation. These were deemed essential for alleviating stammer‐related anxiety and facilitating better speech management. As noted above, adapted interview techniques such as those developed for autistic witnesses may be beneficial (Maras et al., 2020); however, the current findings also underscore the importance of positive interpersonal dynamics between interviewers and interviewees, which extend beyond verbal communication. Indeed, when interviewers built rapport and demonstrated positive body language, patience and understanding, participants reported that they felt better able to provide testimony due to reduced fear of negative reactions (see also Connery et al., 2020). Some participants also suggested that stammer disclosure would be particularly beneficial to their ability to provide testimony by helping to set listener expectations and reducing the pressure they feel to employ these avoidance strategies (Young et al., 2022). However, other PWS are reluctant to report their condition for fear of discrimination (Butler, 2013). Yet the current findings highlight that their perceived likeability, capability and trustworthiness are significantly improved when their difficulties are disclosed. Future research should examine the effect of diagnosis disclosure by PWS not only on how credible they appear, but also on the testimony they subsequently provide in the knowledge that observers are aware of their stammer.

Participants called for increased awareness and education within the CJS to challenge stereotypes and mitigate prejudices that may impact listener perceptions. They also suggested that alternative mediums of testimony should be offered, such as video conferencing, written statements and pre‐recorded testimony at court, to enhance accessibility and reduce anxiety around listener perceptions (Constantino et al., 2022), and that familiarization with the judge and courtroom would be particularly beneficial at trial. These suggestions echo those suggested in the Equal Treatment Bench Book (Judicial College, 2024) and are in line with the Special Measures that are available for vulnerable witnesses under part two of the Youth Justice and Criminal Evidence Act 1999. Special Measures permit adaptations to how evidence is gathered and presented in criminal courts in England and Wales, including screening the witness from the accused, giving evidence by live link or in private, removal of wigs and gowns while the witness gives evidence, video‐recorded evidence‐in‐chief, video‐recorded cross‐examination and re‐examination, evidence given through an intermediary and the use of aids to communications. A judge may also order any non‐statutory ‘extra’ special measures, where appropriate (see Cooper & Mattison, 2017).

Findings also have wider implications for the participation of individuals with communication disorders in CJS proceedings. Considering the importance of juror perceptions of credibility for trial outcomes, calls for routine assessments to identify language difficulties amongst those entering the youth justice system (e.g. Bryan et al., 2015; Winstanley et al., 2021) could be extended to those providing evidence more broadly.

### Limitations

It is important to note limitations of the current research. First, the PWS group in Study 1 was interviewed by an interviewer who already knew of their stammering status. Given that self‐monitoring often arises from the concerns about the perspectives of listeners (Jackson et al., 2021), this may have allowed the PWS witnesses to focus more effectively on the recall task. Future studies involving mock jurors could provide insights into how the presence of evaluators (e.g. police officers, jurors and judges) would impact the performance of PWS.

Second, the testimony interview was conducted virtually, and although the structure mirrored a real‐life interview, it would not have been as cognitively or emotionally demanding (Bagnall & Maras, 2025). Additionally, findings may reflect the views of a niche sample of PWS, given the high chance of selection bias, since the people who volunteered to participate were likely at a specific point with their stammer where they felt confident to do so. Resultantly, the findings may not reflect the views of the broader stammering population, given the diversity of stammering experiences (Tichenor et al., 2022).

Third, there was a baseline age and gender difference between the PWS and comparison groups in Study 1, with the PWS group being slightly older and proportionally more male, which previous evidence indicates may have an effect on eyewitness recall (e.g. Aizpurua et al., 2009; Areh, 2011; Maeder et al., 2012; West & Stone, 2013). It is worth noting, though, that the current PWS group was significantly younger than that of the older adults included in previous studies (Aizpurua et al., 2009; West & Stone, 2013). Groups in the current study were also well matched on cognitive ability. Nevertheless, it is important for future research to replicate these findings with more closely age‐ and gender‐matched groups.

## CONCLUSION

Findings from the current research provide unique insight into how stammering impacts individuals' capacity to provide credible eyewitness testimony. The high stakes and communicative pressures of the testimony context heightened participants' anxiety which, in turn, exacerbated their self‐reported stammer severity and reduced their perceived confidence. A systemic approach to accommodating PWS that considers individual needs in implementation is crucial to ensure PWS can provide meaningful testimony. Such changes may include educating members of the CJS, offering flexible modes of communication and fostering supportive interviewer–interviewee dynamics, while mindful of individual coping strategies. Future research should explore the impact of implementing accommodations in real‐world settings; specifically in terms of effectiveness in improving the experiences of PWS and the quality of their testimony, as well as the impact on legal decision‐making. It would also be helpful to extend this research to suspects and defendants who stammer, where individuals with speech and language difficulties are over‐represented and face disproportionately negative outcomes (e.g. Anderson et al., 2016; Bryan et al., 2015; McNamara, 2012; Winstanley et al., 2021).

## AUTHOR CONTRIBUTIONS

**Katie Maras:** Conceptualization; investigation; writing – original draft; funding acquisition; writing – review and editing; supervision; methodology; formal analysis; project administration; data curation. **Sohee Park:** Conceptualization; investigation; writing – original draft; methodology; formal analysis; data curation; project administration; writing – review and editing. **Patrick Grafton:** Conceptualization; writing – original draft; investigation; methodology; writing – review and editing; formal analysis; project administration; data curation. **Jasmin Peat:** Conceptualization; investigation; writing – original draft; methodology; writing – review and editing; formal analysis; project administration; data curation. **Navyaa Toshniwal:** Data curation; conceptualization; investigation; writing – original draft; methodology; writing – review and editing; formal analysis; project administration. **Alice Haigherty:** Methodology; project administration; investigation. **Kevin Guo:** Investigation; methodology; project administration. **Monty Franks:** Investigation; methodology; project administration. **Hannah Goodwin:** Investigation; methodology; project administration. **Victoria Grau Sainz:** Investigation; methodology; project administration. **Amaira Sharma:** Investigation; methodology; project administration. **Alisa Fridman:** Investigation; methodology; project administration. **Luke Gordon‐Ellis:** Investigation; methodology; project administration. **Kirsten Howells:** Conceptualization; writing – review and editing; investigation.

## Supporting information


Appendix S1

## Data Availability

The data that support the findings of this study are openly available in OSF at https://doi.org/10.17605/OSF.IO/2QNMS.

## Footnotes

^1^
Groups in this trimmed sample did not differ on gender (*p* = .075) or ethnicity (*p* = .273). However, as with the wider sample, the PWS group were significantly older than the comparison group (*p* = .032), albeit with a smaller effect size (*d* = 0.72) than that of Study 1 (*d* = 1.09). As per Study 1, PWS in the trimmed sample still recalled fewer correct details than the comparison group in their free recall (*p* = .003) but were no less accurate (*p* = .234).
