# Supplementary material for: Eyewitness testimony by individuals who stammer: Evidence, experience and perceived credibility
Source: Br J Psychol. 2025 Jul 29;117(1):76–103. doi: 10.1111/bjop.70014 (PMC12783885; doi:10.1111/bjop.70014)
Supplement: Supplementary file 1 — Appendix S1 [file BJOP-117-76-s001.docx]

**Supplementary Material A**

**Qualitative Subjective Experience Survey for Study 1b**

**Opening Questions:**

1. Briefly (i.e., in a few words or a couple of sentences), how does your experience with stammering affect your interactions with others in your day-to-day life?

**Main Questions:**

*Section 1: Remembering the Event*

How easy/challenging did you find **remembering** details of the event?

(Likert scale 1-7)

1. Please explain your answer:
2. What did you find easy (if anything), and why?
3. What did you find challenging (if anything), and why?

To what extent do you feel that your stammer impacted your ability to **remember** details of the event?

(Likert scale 1-7)

1. Please explain your answer.
2. In what ways (if any) did your stammer impact your ability to remember details of the event?

*Section 2: Articulating the Event*

How easy/challenging did you find **articulating** details of the event?

(Likert scale 1-7)

1. Please explain your answer.
2. What did you find easy (if anything), and why?
3. What did you find challenging (if anything), and why?

To what extent do you feel that your stammer impacted your ability to **articulate** details of the event?

(Likert scale 1-7)

1. Please explain your answer:
2. In what ways (if any) did your stammer impact your ability to articulate details about the event?
3. To what extent did you say what you wanted to say during the interview process?

*Section 3: Cognitive and Emotional Impact*

1. Please explain how you felt **before** the interview process (e.g., nervous, excited, neutral)
2. What impact, if any, do you feel that these feelings had on your ability to communicate effectively?
3. Please explain how you felt **during** the interview process.
4. What impact, if any, do you feel that these feelings had on your ability to communicate effectively?

Did you encounter any difficulties in organising your testimony?

[Yes/No?]

1. If yes, can you describe the nature of these difficulties and how you tried to manage them?
2. To what extent did having to provide an eyewitness testimony impact the severity of, or your ability to cope with, your stammer?

*Section 4: Interaction with the Interviewer*

To what extent did you feel worried before the interview about how the interviewer might react to your stammer?

(Likert scale 1-7)

1. Please explain your answer.
2. In what ways (if any) did your expectations of the interviewer’s reaction impact your ability to remember and articulate event details?

To what extent do you feel that the interviewer’s **actual** reaction to your stammer impacted your recall of the event?

(Likert scale 1-7)

1. Please explain your answer.
2. In what ways (if any) did the interviewer’s reaction impact your recall?

*Section 4: Coping Strategies*

Did you use any strategies to manage your speech while providing your eyewitness testimony?

[Yes/No]

1. Please explain your answer.
2. What strategies (if any) did you use?
3. What is it about these strategies that you found helpful?

*Section 5: Suggestions for Improvement*

1. Is there anything else that could have been done to facilitate your recall or improve your experience of giving an account?

**Closing Questions:**

1. Is there anything else you would like to share about your experience of providing an eyewitness testimony as a person who stammers?

**Supplementary Material B**

**Semi-structured qualitative follow-up interview schedule for Study 1b**

**Open questions:**

1. How were you feeling before the eyewitness testimony interview started?
2. PROMPT: How did you feel when you thought about having to provide testimony?
3. PROBE: Had you made any plans or strategies for the interview?
4. PROBE: How do you think that your previous experiences with stammering impacted your mindset prior to your eyewitness testimony interview?

**Main body questions:**

**Section 1: Remembering the Event**

1. In what ways, if any, did your stammer impact your ability to remember details of the event?
   1. PROMPT: To what extent do you feel that managing your stammer interfered with your ability to remember details?
   2. PROBE: Can you elaborate on how your stammer influenced your memory recall during the interview?

**Section 2: Articulating the event**

1. In what ways, if any, did your stammer impact your ability to articulate details of the event?
   1. PROMPT: How did you feel when trying to articulate details and manage your stammer at the same time?
   2. PROBE: Can you describe any specific difficulties you faced while trying to articulate the details of the event?

**Section 3: Cognitive and Emotional Impact**

1. How did you feel during the interview process?
2. PROBE: What impact, if any, did these feelings have on your ability to provide testimony? *[State the feeling the participant mentioned].*
3. To what extent did having to provide an eyewitness testimony impact the severity of, or your ability to cope with, your stammer?
   1. PROBE: Were there any specific factors during the interview that influenced your ability to manage your stammer?

**Section 4: Interaction with the Interviewer**

1. In what ways, if any, did your interaction with the interviewer impact your ability to provide testimony?
   1. PROMPT: Think about their behaviours and/or demeanour.
   2. PROBE: Is there anything you would have liked the interviewer to do differently?

**Section 5: Coping Strategies**

1. What strategies, if any, did you use to manage your speech while providing your eyewitness testimony?
   1. PROBE: What aspects of these strategies did you find particularly helpful?

**Section 6: Suggestions for Improvement**

1. How do you think your testimony would be perceived by a jury?
   1. PROMPT: In what ways, if any, do you think that your stammer might influence a jury’s view of your reliability?
   2. PROBE: How do you feel about the possibility that your stammer might affect the jury’s perception of your testimony?
2. To what extent do you feel that the experience of providing an eyewitness testimony differs between people who stammer and those who do not?
   1. PROBE: What unique challenges do you think that people who stammer face when providing eyewitness testimony?
3. What changes, if any, do you think should be made to facilitate the eyewitness testimony accounts of people who stammer?
   1. PROMPT: What changes could be made to ensure that people who stammer are treated equitably in the legal process?

**Closing questions/ Remarks:**

1. How do you think that your experience in this study would compare to a real-life setting?
   1. PROMPT: In what ways, if any, do you think that your stammer would be affected differently in a real-life setting compared to this study?
2. Is there anything that you think we should have covered today but haven’t yet?

**Supplementary Material C**

**Additional exemplary quotes for each theme and sub-theme in Study 1b**

| **Theme** | **Sub-theme** | **Codes** | **Additional exemplary quotes** |
| --- | --- | --- | --- |
| **1. A vicious cycle of anxiety, pressure, and stammering** |  | - Providing testimony is especially anxiety-provoking for PWS. - Speaking in front of multiple people increases stammer severity. - Increased stammering severity in high-pressure situations. - Stammer severity influenced by anxiety/stress. - Pressure of real-life consequences increase stammer severity. - Nervousness exacerbates stammering. | *“I believe stammering’s all through the fear of stammering. So, you would be hearing stammering even more because you were in a position of authority and under pressure so … umm … so you’re even more likely to stammer anyway”* (Oliver, 44, male).  *“If I’m worried about what I’m going to say is going to have an impact on, you know, potentially others, my stammer becomes more uncontrollable”* (Kaya, 32, female).  *“Especially in a group situation or to a lot of strangers at one time, and I would be nervous about it the whole week building up to it”* (Jamie, 54, male). |
|  | Worries about perceived credibility | - Insufficient understanding of stammering. - Concern that stammering behaviours may be misinterpreted as dishonesty. - Concern over impact of prejudice on listener’s perceptions of testimony reliability and credibility. - Embarrassment about stammering affects confidence. | *“[…] for people who don’t stammer it’s more what they’re saying but for people who stammer we have to think about, you know, how we say it and also … like if we do stammer, what impact that will have on listeners.”* (Ben, 24, male).  *“I’ve heard that apparently … if people stammer or stumble, or they’re, you know, sort of, you know, umming and ahhing, it means that they’re probably lying, but that’s not necessarily true, because if somebody has a stammer and you put them in a high-pressure situation, what would you expect that, you know, the stammer’s going to get even worse.”* (Matthew, 29, male).  *“I would be very worried in real life about providing eyewitness testimony as I would think that people would automatically think I was lying or making it up and that I was a terrible witness. I would feel useless in a real-life crime investigation.”* (Sophia, 31, female).  *“I know what I’m saying is correct and I am saying it, and I believe it like with all my heart, but how I’m articulating it is making me look as though I’m uncredible, I don’t know what I’m saying”* (Kaya, 32, female).  *“I think as a person who stammer we are on the back foot as reliable and credible witnesses and the stereotypes of people who stammer exacerbates this”* (Logan, 59, male). |
| **2. Impression management** |  | - Preoccupation with concerns about listener perceptions due to stammer. - Anxiety about listener’s reactions to stammer. - Preoccupation with self-consciousness over outward appearance to listeners. - Focus on act of speaking over testimony content. - Constant vigilance to avoid stammering. - Testimony detail is limited to avoid stammering. - Word switching may compromise the clarity and precision of testimony. - Speech output is altered to avoid words that induce stammering. - Avoiding eye contact aids stammer management. | *“You’re always in two minds as a person who stammers. So, like you know, you’re very fixed ... or like your verbal fluency and like how you’re being interpreted by the listener and also like kind of like you’re listening for their feedback ... you know ... to see if it’s positive or negative”* (Ben, 24, Male).  *“I’ve got a lot of techniques and coping mechanisms to speak how I’m speaking to you right now and it involves me constantly interchanging words and letters that I know I can’t say properly”* (Kaya, 32, female).  *“It’s difficult to explain something in more detail because I always look for ways to keep answers short while articulating something”* (Ruby, 32 female).  *“[…] like changing your eye contact so that you can kind of focus on something else and restart”* (Kaya, 32, female). |
|  | Collateral damage | - The significant effort and energy required to speak fluently is exhausting. - Stammering does not impact memory recall. - Stammering does not prevent expression of all remembered details. - Stammering affects expression rather than memory. - Discrepancy between intended and spoken testimony may impact accuracy. | *“You’d still be able to get the message across … but not as … to the point, I guess. So … if … I don’t know, we were talking about the bar scene, and I couldn’t get the word bar out I’d have to go all the way around, switching to another word and people would just think, what’s going on? You know? So, you could still get the message across … but it’s not as succinct, to the point because we’re having to go around the houses”* (William, 45, male).  *“My stammer has nothing to do with the way I think or the way I gather* *information, it doesn’t have anything to do with it”* (Jacob, 30, male). |
| **3. Levelling the playing field: Ensuring accessible testimony** |  | - Need to extend accommodations to PWS to make testimony process equitable, like for other types of disabilities. - Adaptations should consider individual needs. - Performance in testimony interview depends on where an individual is in their stammering journey. - Unpredictability of stammer. | *“We wear glasses, people who are hard of hearing wear a hearing aid, but for people who stammer it’s not easy to articulate and it’s not easy to do something about that, so I think adaptations should be made and awareness … awareness needs to be spread”* (William, 45, male).  *“I do think that reflexes to where I’m at in kind of my stammering journey, so like 15 years ago I wouldn’t have even agreed to it so umm … I can imagine that’s going to be quite individual”* (Oliver, 44, male).  *“I think it depends on the level of the person that has a stammer and what kind of techniques and things that they have in place”* (Kaya, 32, female). |
|  | Individual and systemic facilitators | - Slowing speech and pausing are effective strategies for managing stammer during testimony. - Having time facilitates testimony expression. - Remaining calm and relaxed facilitates question answering. - Preparation helps to manage stammer. - Disclosing stammer to set listener expectations reduces pressure. - Disclosure of stammer needed to prevent misinterpretations of stammering behaviours. - Allowing written responses as an option to bypass verbal barriers. - Providing testimony through alternative mediums to minimise the pressure of the environment. - Need for awareness and understanding of stammering by individuals involved in the criminal justice system. - Need to mitigate the impact of prejudices through education. - One-to-one communication is easier. | *“I slowed down my speech so that I could be more articulate. It is helpful since talking at a slower pace relaxes me” (Bea, 23, female).*  *“Maybe at least rehearse or practice to talk or say some answers, you know, what kind of answers I would … sorry … what kind of questions I would expect or probably try to practice the way I would answer these things” (Ruby, 32, female).*  *“[…] if in these circumstances someone introduced you to the court as having a stammer that fear reduces a lot. So, one of the recommendations for anything … wherever I go, but this would be particularly … relevant, is when you’re introduced as having a stammer you don’t have that worry of ‘oh I can’t stammer because they might find out’, and that would really help” (William, 45, male).*  *“If there was a video link set up even in a room next door, so you, you’re just seeing one person, but everyone else can see you on a screen that would help massively”* (William, 45, male).  *“I don’t know whether you could do a written thing instead as an option, or you could certainly ask that individual, would you prefer to write this down”* (Oliver, 44, male).  *“I think it would be important for a jury to have a full understanding of what a stammer is and also like ... yeah ... and as .... you know many walks of life people aren’t really aware or educated as to what a stammer is”* (Ben, 24, male). |
|  | The crucial role of the interviewer | - Supportive interviewer demeanour enhances fluency and reduces anxiety. - Negative interviewer demeanour exacerbates stammering severity and impairs communication. - Interviewer patience facilitates providing testimony. - Reassurance from interviewer would facilitate testimony. - Negative impact of listener impatience on fluency and confidence. - Familiarisation with person in authority facilitates providing testimony. - Interaction with unfamiliar individual increases stammer severity. - Importance of trust and rapport in communicating with the interviewer. | *“Interviewer’s facial expression was neutral, thank you. If that was not neutral and I observed something like a frown, or crease in the eyebrow area, I would then stop looking at them directly. The possibility of my stammering becoming more evident would be pronounced” (Talia, 47, female).*  *“If it was just some, you know, person I’ve never met in my life asking me all these questions, then even that, you know what I mean would massively impact my ability to articulate what I’m trying to say” (Matthew, 29, male).*  *“Speak with myself, sort of get to know my situation just so we can build at least a bit of rapport and trust because you know having the trust is a massive thing” (Matthew, 29, male).* |

**Supplementary Material D**

**Witness Credibility Scale (adapted from Brodsky et al., 2010) for Studies 2 and 3**

| 1 | 2 | 3 | 4 | 5 | 6 | 7 | 8 | 9 | 10 |
| --- | --- | --- | --- | --- | --- | --- | --- | --- | --- |
| Unfriendly |  |  |  |  |  |  |  |  | Friendly |
| 1 | 2 | 3 | 4 | 5 | 6 | 7 | 8 | 9 | 10 |
| Disrespectful |  |  |  |  |  |  |  |  | Respectful |
| 1 | 2 | 3 | 4 | 5 | 6 | 7 | 8 | 9 | 10 |
| Unkind |  |  |  |  |  |  |  |  | Kind |
| 1 | 2 | 3 | 4 | 5 | 6 | 7 | 8 | 9 | 10 |
| Ill-mannered |  |  |  |  |  |  |  |  | Well-mannered |
| 1 | 2 | 3 | 4 | 5 | 6 | 7 | 8 | 9 | 10 |
| Unpleasant |  |  |  |  |  |  |  |  | Pleasant |
| 1 | 2 | 3 | 4 | 5 | 6 | 7 | 8 | 9 | 10 |
| Untrustworthy |  |  |  |  |  |  |  |  | Trustworthy |
| 1 | 2 | 3 | 4 | 5 | 6 | 7 | 8 | 9 | 10 |
| Untruthful |  |  |  |  |  |  |  |  | Truthful |
| 1 | 2 | 3 | 4 | 5 | 6 | 7 | 8 | 9 | 10 |
| Undependable |  |  |  |  |  |  |  |  | Dependable |
| 1 | 2 | 3 | 4 | 5 | 6 | 7 | 8 | 9 | 10 |
| Dishonest |  |  |  |  |  |  |  |  | Honest |
| 1 | 2 | 3 | 4 | 5 | 6 | 7 | 8 | 9 | 10 |
| Unreliable |  |  |  |  |  |  |  |  | Reliable |
| 1 | 2 | 3 | 4 | 5 | 6 | 7 | 8 | 9 | 10 |
| Not confident |  |  |  |  |  |  |  |  | Confident |
| 1 | 2 | 3 | 4 | 5 | 6 | 7 | 8 | 9 | 10 |
| Inarticulate |  |  |  |  |  |  |  |  | Well-spoken |
| 1 | 2 | 3 | 4 | 5 | 6 | 7 | 8 | 9 | 10 |
| Tense |  |  |  |  |  |  |  |  | Relaxed |
| 1 | 2 | 3 | 4 | 5 | 6 | 7 | 8 | 9 | 10 |
| Shaken |  |  |  |  |  |  |  |  | Poised |
| 1 | 2 | 3 | 4 | 5 | 6 | 7 | 8 | 9 | 10 |
| Not self-assured |  |  |  |  |  |  |  |  | Self-assured |
| 1 | 2 | 3 | 4 | 5 | 6 | 7 | 8 | 9 | 10 |
| Incomplete account |  |  |  |  |  |  |  |  | Complete account |
| 1 | 2 | 3 | 4 | 5 | 6 | 7 | 8 | 9 | 10 |
| Unclear account |  |  |  |  |  |  |  |  | Clear account |
| 1 | 2 | 3 | 4 | 5 | 6 | 7 | 8 | 9 | 10 |
| Uneducated |  |  |  |  |  |  |  |  | Educated |
| 1 | 2 | 3 | 4 | 5 | 6 | 7 | 8 | 9 | 10 |
| Incapable of testimony |  |  |  |  |  |  |  |  | Capable of testimony |
| 1 | 2 | 3 | 4 | 5 | 6 | 7 | 8 | 9 | 10 |
| Inaccurate account |  |  |  |  |  |  |  |  | Accurate account |

**Supplementary Material E**

**Adapted Version of the ‘Public Speaking Scale’ and Item Descriptors (Study 2)**

***Point 1 - Projection***

*Witness should not be speaking too loudly or too softly. Should be loud enough for you to hear clearly. Should be a little louder than normal conversational voice. Projection should be varied at times of emphasis.*

| **Item No.** | **Description** |
| --- | --- |
| **5** | Witness spoke loud enough so that you could easily hear. Projection was sometimes louder or softer to emphasize important details. |
| **4** | Witness spoke loud enough, but was too loud or too soft a few times during the statement. Witness tried to use variation in projection for emphasis but was awkward. |
| **3** | The witness generally spoke loud enough, but sometimes may be too loud or too soft for easy listening. No variation in projection to emphasize important points. |
| **2** | Witness generally did not speak loud enough for easy listening. However, it was possible with effort to hear the points being made. No variation in projection for emphasis. |
| **1** | Witness could not be understood without great effort by you. |

***Point 2 - Pace***

*Witness should not speak too quickly or too slowly. Should be at a good speed for you to hear all points made. Speed should be varied at times of emphasis.*

| **Item No.** | **Description** |
| --- | --- |
| **5** | Witness spoke at a good pace, so that you could easily hear and comprehend. Pace was sometimes faster or slower to emphasize important points. |
| **4** | Witness used a good pace, but was too fast or too slow a few times during the presentation. Witness tried to use variation in pace for emphasis, but was awkward. |
| **3** | Witness generally spoke at a good pace, but sometimes may be too fast or too slow for easy listening. No variation in pace to emphasize important points. |
| **2** | Witness generally did not speak at a good pace for easy listening. However, it was possible with effort to hear the points being made. No variation in pace for emphasis. |
| **1** | Witness could not be understood without great effort by you. |

***Point 3 - Intonation***

*Witness should be speaking smoothly in phrases, so that event details are clear. Intonation is very important for being a good speaker. Pitch patterns help clarify the type of information and its importance. Use pauses to make each point clear.*

| **Item No.** | **Description** |
| --- | --- |
| **5** | Witness spoke smoothly so that you could easily hear. Pitch patterns and pauses were used effectively to emphasize important points. |
| **4** | Witness spoke smoothly enough, but had a few awkward pauses during the presentation. Witness tried to use variation in pitch to emphasize important points, but was awkward. |
| **3** | Witness generally spoke smoothly enough but sometimes may have awkward or long pauses. No variation in pitch or use of pauses to emphasize important points. |
| **2** | Witness generally did not speak smoothly enough for easy listening. It was possible with effort to hear the points being made. No variation in pitch for emphasis. |
| **1** | Witness could not be understood without great effort by you. |

***Point 4 - Diction***

*All words/phrases should be clearly spoken so that you can easily hear/understand the witness’s points.*

| **Item No.** | **Description** |
| --- | --- |
| **5** | Witness spoke clearly so that you could easily hear. All key words were clear. Accent and syllable stress were used effectively during the statement. |
| **4** | Witness spoke smoothly enough, but had a few words that were unclear. Witness tried to use accent and syllable stress, but was awkward. |
| **3** | Witness generally spoke clearly enough but sometimes may have had accent or syllable stress. Overall meaning could be understood, but some words were unclear. |
| **2** | Witness generally did not speak clearly enough for easy listening. It was possible with effort to hear the points being made. Many words were not understood. |
| **1** | Witness could not be understood without great effort by you. |

***Point 5 - Language Use***

*The witness’s sentences should be clear and describe the event. Emphasize important points by using short clear sentences and some repetition.*

| **Item No.** | **Description** |
| --- | --- |
| **5** | All event details were clearly expressed, sentence structures were varied, and there was some repetition for important points. There were no serious errors that hindered comprehension. |
| **4** | Most event details were clearly expressed, sentence structures were varied, and there was some repetition for important points. There were few errors that hindered comprehension. |
| **3** | In general, event details were clearly expressed, sentence structures were varied, and there was some repetition/summary for important points. There were some serious errors. |
| **2** | Most event details were not clearly expressed, sentence structures were not often varied, and there was no repetition for important points. There were many serious errors. |
| **1** | Witness was nearly incomprehensible due to structural errors that hindered understanding. |

***Point 6 - Vocabulary***

*The witness should use appropriate and understandable language, being careful of word choice and word forms.*

| **Item No.** | **Description** |
| --- | --- |
| **5** | Vocabulary usage was always appropriate or understandable for the jury. No serious errors in word choice or word forms. |
| **4** | Vocabulary usage was mostly appropriate or understandable for the jury. A few serious errors in word choice or word forms. |
| **3** | Vocabulary usage was not always appropriate or understandable for the jury. Some serious errors in word choice or word forms. |
| **2** | Vocabulary usage was often not appropriate or understandable for the jury. Many serious errors in word choice or word forms. |
| **1** | Witness was nearly incomprehensible due to serious errors in word choice or word forms. |

***Point 7 – Persuasiveness***

*Witness’s statement should be compelling: informing and persuading the jury*

| **Item No.** | **Description** |
| --- | --- |
| **5** | The witness’s statement was persuasive and compelling, leading you to believe it. |
| **4** | The witness’s statement was more or less persuasive and compelling, but had a few weak sections. |
| **3** | The witness’s statement was somewhat persuasive and compelling, but had some weak parts. |
| **2** | The witness’s statement was mostly not persuasive and compelling, but included some good points during the testimony. |
| **1** | The witness’s statement was not persuasive and compelling at all. |

**Supplementary Material F**

**Stammering Information (Studies 2 and 3)**

Stammering (also known as stuttering) is a difference in the way some people speak. Stammering makes it difficult for the person to produce a smooth flow of speech. Stammering most commonly begins between the ages of 2-5, although it can also begin later including in adulthood. Onset can be gradual or sudden. Features vary from person to person as well as over time, but can include repetition of sounds and words, elongation of sounds, and blocking where no sound comes out even though the person is trying to speak. You might sometimes see physical or facial effort as the person works hard to push out a word. Although everyone may experience moments of dysfluent speech occasionally, it happens much more often for people who stammer, sometimes nearly every time they speak. Stammering can also take an emotional and cognitive toll. Individuals may fear particular words and situations due to stammering and try to avoid them in order to hide their stammering from others. Previous research suggests around 1% of the adult population stammer, but recent work indicates the true figure may be higher.

**Supplementary Material G**

**Knowledge of Stammering Questionnaire (Study 3)**

Answered: True, False or Unsure

1. Stammering is also known as stuttering
2. Stammering can involve someone repeating sounds or words
3. Stammering can involve someone stretching or prolonging sounds
4. Stammering can involve someone experiencing a silent block in their speech where a sound gets stuck
5. Stammering is always obvious
6. Stammering always develops during childhood
7. Stammering is more common amongst males than females
8. People who stammer are legally protected against disability discrimination under the Equality Act and Disability Discrimination Act in the UK
9. People who stammer can adopt strategies to appear more fluent in social situations
10. People who stammer do not often report difficulties in schooling, work and building relationships because of their speech challenges
11. Stammering can induce high levels of anxiety
12. Some people who stammer sometimes avoid speaking situations
13. People who stammer often find it difficult to participate in the criminal justice system.
14. Individuals who stammer are no more likely to receive an unfair sentence than those without speech difficulties
15. When giving testimony, a person who stammers is no more likely to be perceived as dishonest as an individual without a speech difficulty
